# Vascular Endothelial Growth Factor Genotypes and Haplotypes Contribute to the Susceptibility of Obstructive Sleep Apnea Syndrome

**DOI:** 10.1371/journal.pone.0114582

**Published:** 2014-12-26

**Authors:** Chao Cao, Qunli Ding, Dan Lv, Zhe Dong, Shifang Sun, Zhongbo Chen, Huahao Shen, Zaichun Deng

**Affiliations:** 1 Department of Respiratory Medicine, Affiliated Hospital of School of Medicine, Ningbo University, Ningbo, 315020, China; 2 Department of Respiratory and Critical Care Medicine, Second Affiliated Hospital, Zhejiang University School of Medicine, Hangzhou, 310009, China; 3 Zhejiang University School of Medicine, Hangzhou, 310058, China; Charité - Universitätsmedizin Berlin, Germany

## Abstract

**Background:**

To investigate whether *VEGF* polymorphisms (*-460T/C, +405G/C*, and *+936C/T*)/haplotypes influence the susceptibility of obstructive sleep apnea (OSA).

**Method:**

A prospective case-control study was conducted to evaluate the genetic effects of *VEGF* polymorphisms on the development of OSA. 150 patients and 225 healthy controls were recruited for this study and their genotypes were determined by polymerase chain reaction and restriction fragment length polymorphism (PCR-RFLP). The odds ratios (OR) and 95% confidence intervals (CI) were calculated by logistic regression analysis.

**Result:**

Our study showed that the *-460C* allele (C *vs.* T: OR = 1.95, 95% CI = 1.38–2.76) and *+936T* allele (T *vs.* C: OR = 1.48, 95% CI = 1.02–2.15) were associated with an increased OSA risk, whereas *+405C* allele was associated with a decreased susceptibility to OSA (C *vs.* G: OR = 0.61, 95% CI = 0.45–0.83). Compared with the most common haplotype *CCT*, *CGC* (OR = 2.22, 95% CI = 1.19–4.13) and *TGC* (OR = 3.83, 95% CI = 1.56–9.40) were associated with a significantly increased risk of OSA.

**Conclusion:**

These observations implied that *VEGF* gene polymorphisms might be associated with the susceptibility to OSA. These results need to be validated by other independent studies, especially in diverse ethnic populations.

## Introduction

Obstructive sleep apnea (OSA) syndrome is a respiratory disorder characterized by a unique form of intermittent hypoxia, with short, repetitive cycles of hypoxia and reoxygenation [1]. One of the physiologic adaptive responses of tissue to concomitant episodes of systemic hypoxia is angiogenesis [2]. Hypoxia is a major stimulator of vascular endothelial growth factor (VEGF) expression [3]. It also induces the activation of hypoxia inducible factor (HIF-1), which is a cellular hypoxia sensor and a key element in the process of oxygen homeostasis and in the regulation of VEGF mRNA transcription [4]. Extensive studies have provided evidences that serum levels of VEGF are elevated in patients with OSA related to the degree of nocturnal oxygen desaturation [5]–[9].

The *VEGF* gene is located on chromosome 6p21.3 and comprises a family of six protein isoforms (VEGF-A, VEGF-B, VEGF-C, VEGF-D, VEGF-E, and placenta growth factor [PlGF]) [10], [11]. In past decades, numbers of single-nucleotide polymorphisms (SNPs) have been identified and widely studied in the VEGF gene. Among these, the common three *VEGF* SNPs, *-460T/C* (rs833061) and *+405G/C* (rs2010963) in the 5′-untranslated region and *+936C/T* (rs3025039) in the 3′-untranslated region, were found to be associated with differential VEGF expression and involved in many kinds of disorders in which angiogenesis was critical in the development of disease [12], [13]. In our previous studies, we observed their potential role in modifying the susceptibility to pulmonary diseases [14], [15].

Recently, numerous epidemiological studies have described several genetic variants as biomarkers for genetic susceptibility to OSA development [16]–[20]. Moreover, in our previous study, we even observed a functional *EGF+61A/G* polymorphism was associated with the severity of OSA [21]. Nevertheless, the relationship between *VEGF* polymorphisms and risk of OSA has not been evaluated worldwide. Based on the pathologic significance of *VEGF* in OSA and the potential biological effects of *VEGF* polymorphisms on VEGF expression, we hypothesized that some functional polymorphisms of the *VEGF* gene would be associated with differential risk of OSA. Thus, we conducted a case-control study to investigate whether *VEGF* polymorphisms (*-460T/C, +405G/C, and +936C/T*)/haplotypes influence the susceptibility of OSA.

## Methods

### Study subjects

A prospective case-control study was conducted in Respiratory Medicine of Affiliated Hospital of Ningbo University in China from October 2011 to August 2013. Apnea–hypopnea index (AHI) was measured by overnight polysomnography (PSG) studies. OSA was defined as an AHI >5 events/h, and daytime symptoms specific for an OSA syndrome. The severity of OSA was graded on the basis of AHI, with suggested thresholds for mild (AHI ≥5 and <15), moderate AHI ≥15 and <30), and severe OSA (AHI ≥30) [22]. The healthy control group was composed of subjects of the same ethnic origin as the patients. The control subjects were included on the basis of the following criteria: absence of sleep disturbance, no daytime sleepiness, and AHI <5 events/h. Enrolled patients and controls were born and were residing in Southeast of China.

All study participants had to complete a questionnaire to elicit information on demographic features, sex, ethnicity, dietary habits, prior disease history, medications, physical activities, tobacco and alcohol use, weight, family history of disease, and an assessment of sleeping habits before PSG. Approval for this study was obtained from the Institutional Review Board for Human Studies of Affiliated Hospital, School of Medicine, Ningbo University (Ningbo, China). All subjects were unrelated Chinese Han individuals and written informed consent was obtained from all participating subjects.

### *VEGF* genotyping assays

The extraction of genomic DNA from the peripheral blood lymphocytes was performed using a commercially available DNA isolation kits (Tiangen, Beijing) following the manufacturer's protocol. Genotypes were determined using polymerase chain reaction-restriction fragment length polymorphism (PCR-RFLP) assay. PCR reactions were done in a 20 µL reaction volume containing 60∼100 ng of extracted DNA, 5 pmol of the each primer which was designed as previously described [23], [24], 200 µmol of dNTP, 1 unit of Taq polymerase, and 2 µL of buffer. PCR was commenced with incubation at 94°C for 5 min, followed by 30 cycles of 94°C for 30 s, 58°C for 30 s (60°C for -460T/C), and 72°C for 30 s. Final extension was done at 72°C for 5 mins. The restriction enzymes for the *-460T/C*, *+405G/C*, and *+936C/T* genotypes were *Bsa* HI, *Bsm* FI, and *Nla* III, respectively. The digested PCR products were resolved on 3% acrylamide gel to obtain genotypes.

### Statistical analysis

The genotypic distribution of alleles at individual loci was tested to compare the observed with expected genotype frequencies for the Hardy–Weinberg equilibrium using the χ2 test. Cases and controls were compared using the Student's t test for continuous variables and the χ2 test for categorical variables. The associations between the VEGF genotypes and risk of OSA were estimated by computing the odds ratios (ORs) and their 95% confidence intervals (CIs) by logistic regression analysis. Linkage disequilibrium coefficients (D′) were calculated using SHEsis software [25]. In addition, the relationship between *-460T/C*, *+405G/C*, and *+936C/T* haplotypes and OSA risk were evaluated, using Haplo Stats package version 1.6.8 in R software version 3.0.3. The statistical analyses were done using the SPSS (version 13.0, Chicago, III., United States) and GraphPad Prism 5.0 (GraphPad Software, San Diego, CA). Statistically significant threshold was *P* value less than 0.05.

## Results

### Patient characteristics

Basic demographic characteristics for cases and controls were summarized in Table 1. Not surprisingly, most patients included in our study were males and overweight, as these are known risk factors for the development of OSA. The mean age of the patients was 48.7 years (range 25–79), and 114 patients (76.0%) were male. Among 225 subjects in the control group (179 males and 49 females), the average age was 47.9±9.5 years (range 27–78). The percentage of smokers in OSA patients and control group was 40.6% and 50.2%, respectively. There was no significant difference in age, gender, and smoking status between two groups. Patients with OSA were divided into 40 mild patients (26.7%), 42 moderate patients (28.0%), and 68 severe patients (45.3%) according to their AHI value.

**Table 1:** Demographics and clinical characteristics of OSA patients and controls.

Variables	OSA patients	Controls
Age (mean ± SD), years	48.7±11.5	47.9±9.5
Sex [male/female ratio]	114: 36	176: 49
BMI [kg/m2]	27.2±3.6	24.9±3.7
Smokers (%)	44.6%	50.2%
AHI [events/h]	33.2±22.1	2.9±0.9
Mild OSA [%]	26.7%	
Moderate OSA [%]	28.0%	
Severe OSA [%]	45.3%	
Hypertension [%]	32.0%	
Diabetes mellitus [%]	10.7%	

OSA  =  Obstructive sleep apnea.

### Associations between *VEGF* genotypes and OSA risk

**Figure 1:**
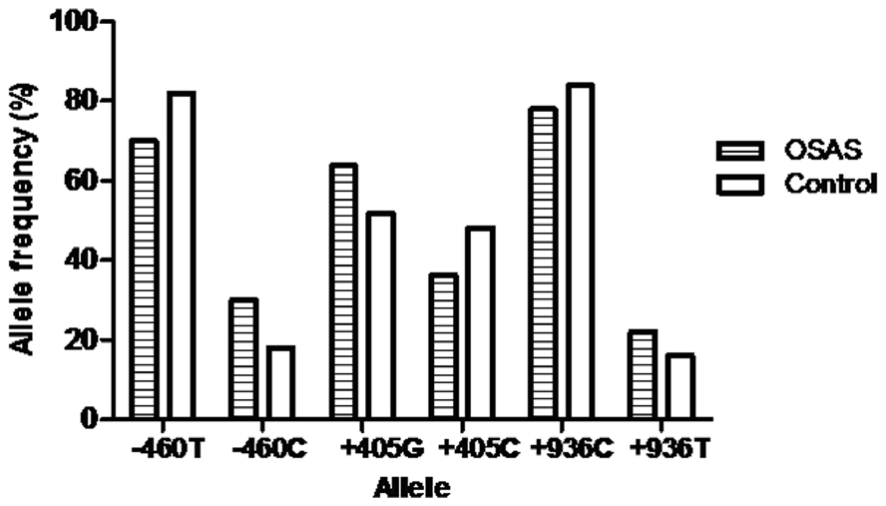
The allele frequencies of three polymorphisms in the VEGF gene in OSA patients and controls.

The genotype and polymorphic allele frequencies of *-460T/C*, *+405G/C*, and *+936C/T* among OSA patients and controls were shown in Table 2 and Fig. 1. All of the *VEGF* polymorphisms in the controls were consistent with Hardy-Weinberg equilibrium (*P*>0.05, χ2 goodness-of-fit). Compared with *-460 TT* genotype, *CC* genotype (OR = 7.79, 95% CI = 2.52–24.11) and *TC/CC* genotype (OR = 1.87, 95% CI = 1.23–2.85) were associated with an increased OSA risk. In addition, statistically significant differences were also observed in the allele comparison (C *vs.* T: OR = 1.95, 95% CI = 1.38–2.76) and recessive model for C allele (CC *vs.* TT/TC: OR = 6.60, 95% CI = 2.16–20.15). For the *+405G/C* polymorphism, the CC genotype was associated with a decreased susceptibility to OSA than those with the GG (OR = 0.40, 95% CI = 0.22–0.73) or GG/GC (OR = 0.51, 95% CI = 0.29–0.87) genotype. Statistically similar results were found in two other genetic models (C *vs.* G: OR = 0.61, 95% CI = 0.45–0.83; CC/GC *vs.* GG: OR = 0.56, 95% CI = 0.36–0.87). For the *+936C/T* polymorphism, a significant difference was found in the allele comparison between OSA patients and controls (T *vs.* C: OR = 1.48, 95% CI = 1.02–2.15). However, no evidence of association between *VEGF +936C/T* polymorphism and risk of OSA was observed as we compared other genetic models (TT *vs.* CC, CT *vs*. GC, CT/TT *vs.* CC, or TT *vs.* CT/TT).

**Table 2:** Genotype distributions and allele frequencies in patients with OSA patients and controls.

Polymorphism		Total (n = 375)		Cases (n = 150)		Controls (n = 225)	*P-*Value		OR (95% CI)	
VEGF -460C/T										
TT		224 (59.7)		76 (50.7)		148 (65.8)			1 (Ref.)	
TC		131 (34.9)		58 (38.7)		73 (32.4)	0.05		1.55 [0.99, 2.41]	
CC		20 (5.3)		16 (10.7)		4 (1.8)	0.0004		7.79 [2.52, 24.11]	
TT+TC		355 (94.7)		134 (89.3)		221 (98.2)			1 (Ref.)	
CC		20 (5.3)		16 (10.7)		4 (1.8)	0.0009		6.60 [2.16, 20.15]	
TT		224 (59.7)		76 (50.7)		148 (65.8)			1 (Ref.)	
TC+CC		151 (40.3)		74 (49.3)		77 (34.2)	0.004		1.87 [1.23, 2.85]	
T allele		579 (77.2)		210 (70.0)		369 (82.0)			1 (Ref.)	
C allele		171 (22.8)		90 (30.0)		81 (18.0)	0.0001		1.95 [1.38, 2.76]	
VEGF +405C/G										
GG		128 (34.1)		63 (42.0)		65 (28.9)			1 (Ref.)	
GC		168 (44.8)		65 (43.3)		103 (45.8)	0.07		0.65 [0.41, 1.04]	
CC		79 (22.1)		22 (14.7)		57 (25.3)	0.003		0.40 [0.22, 0.73]	
GG+GC		296 (78.9)		128 (85.3)		168 (74.7)			1 (Ref.)	
CC		79 (21.1)		22 (14.7)		57 (25.3)	0.01		0.51 [0.29, 0.87]	
GG		128 (34.1)		63 (42.0)		65 (28.9)			1 (Ref.)	
GC+CC		247 (65.9)		87 (58.0)		160 (71.1)	0.009		0.56 [0.36, 0.87]	
G allele		424 (56.5)		191 (63.7)		233 (51.8)			1 (Ref.)	
C allele		326 (43.5)		109 (36.3)		217 (48.2)	0.001		0.61 [0.45, 0.83]	
VEGF +936C/T										
CC		256 (68.3)		94 (62.7)		162 (72.0)			1 (Ref.)	
CT		100 (26.7)		46 (30.7)		54 (24.0)	0.11		1.47 [0.92, 2.34]	
TT		19 (5.1)		10 (6.7)		9 (4.0)	0.17		1.91 [0.75, 4.88]	
CC+CT		356 (94.9)		140 (93.3)		216 (96.0)			1 (Ref.)	
TT		19 (5.1)		10 (6.7)		9 (4.0)	0.25		1.71 [0.68, 4.32]	
CC		256 (68.3)		94 (62.7)		162 (72.0)			1 (Ref.)	
CT+TT		119 (31.7)		56 (37.3)		63 (28.0)	0.06		1.53 [0.99, 2.38]	
C allele		612 (81.6)		234 (78.0)		378 (84.0)			1 (Ref.)	
T allele	138 (18.4)		66 (22.0)		72 (16.0)			0.04		1.48 [1.02, 2.15]

Polymorphism	Total (n = 375)	Cases (n = 150)	Controls (n = 225)	*P-*Value	OR (95% CI)
VEGF -460C/T					
TT	224 (59.7)	76 (50.7)	148 (65.8)		1 (Ref.)
TC	131 (34.9)	58 (38.7)	73 (32.4)	0.05	1.55 [0.99, 2.41]
CC	20 (5.3)	16 (10.7)	4 (1.8)	0.0004	7.79 [2.52, 24.11]
TT+TC	355 (94.7)	134 (89.3)	221 (98.2)		1 (Ref.)
CC	20 (5.3)	16 (10.7)	4 (1.8)	0.0009	6.60 [2.16, 20.15]
TT	224 (59.7)	76 (50.7)	148 (65.8)		1 (Ref.)
TC+CC	151 (40.3)	74 (49.3)	77 (34.2)	0.004	1.87 [1.23, 2.85]
T allele	579 (77.2)	210 (70.0)	369 (82.0)		1 (Ref.)
C allele	171 (22.8)	90 (30.0)	81 (18.0)	0.0001	1.95 [1.38, 2.76]
VEGF +405C/G					
GG	128 (34.1)	63 (42.0)	65 (28.9)		1 (Ref.)
GC	168 (44.8)	65 (43.3)	103 (45.8)	0.07	0.65 [0.41, 1.04]
CC	79 (22.1)	22 (14.7)	57 (25.3)	0.003	0.40 [0.22, 0.73]
GG+GC	296 (78.9)	128 (85.3)	168 (74.7)		1 (Ref.)
CC	79 (21.1)	22 (14.7)	57 (25.3)	0.01	0.51 [0.29, 0.87]
GG	128 (34.1)	63 (42.0)	65 (28.9)		1 (Ref.)
GC+CC	247 (65.9)	87 (58.0)	160 (71.1)	0.009	0.56 [0.36, 0.87]
G allele	424 (56.5)	191 (63.7)	233 (51.8)		1 (Ref.)
C allele	326 (43.5)	109 (36.3)	217 (48.2)	0.001	0.61 [0.45, 0.83]
VEGF +936C/T					
CC	256 (68.3)	94 (62.7)	162 (72.0)		1 (Ref.)
CT	100 (26.7)	46 (30.7)	54 (24.0)	0.11	1.47 [0.92, 2.34]
TT	19 (5.1)	10 (6.7)	9 (4.0)	0.17	1.91 [0.75, 4.88]
CC+CT	356 (94.9)	140 (93.3)	216 (96.0)		1 (Ref.)
TT	19 (5.1)	10 (6.7)	9 (4.0)	0.25	1.71 [0.68, 4.32]
CC	256 (68.3)	94 (62.7)	162 (72.0)		1 (Ref.)
CT+TT	119 (31.7)	56 (37.3)	63 (28.0)	0.06	1.53 [0.99, 2.38]
C allele	612 (81.6)	234 (78.0)	378 (84.0)		1 (Ref.)
T allele	138 (18.4)	66 (22.0)	72 (16.0)	0.04	1.48 [1.02, 2.15]

Values in parentheses represent percentage; VEGF  =  Vascular endothelial growth factor; OSA  =  Obstructive sleep apnea; OR  =  Odds ratio; CI  =  Confidence interval; Ref.  =  Reference.

### Analysis of linkage disequilibrium

Linkage disequilibrium between the three polymorphisms was calculated based on Lewontin's D' in controls. Linkage disequilibrium coefficient (D') was 0.48 for *-460T/C* and *+405G/C*, 0.10 for *-460T/C* and *+936C/T*, and 1.00 for *+405G/C* and *+936C/T*. The corresponding R^2^ (correlation coefficient squared) was 0.04 for *-460T/C* and *+405G/C*, 0.01 for *-460T/C* and *+936C/T*, and 0.19 for *+405G/C* and *+936C/T*.

### *VEGF* haplotype and susceptibility to OSA

**Figure 2:**
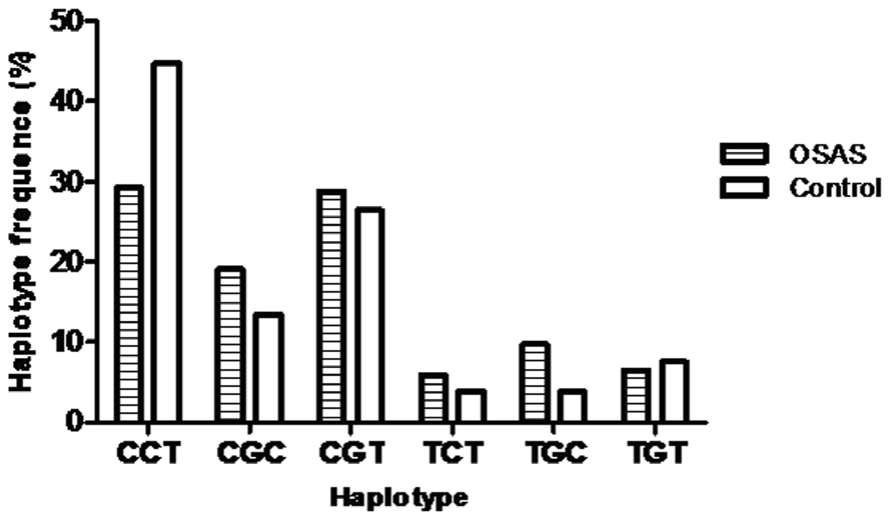
The observed frequencies of the haplotypes of three polymorphisms in the VEGF gene in OSA patients and controls. Nucleotides are listed in the order -460T/C, +405G/C, and +936C/T.

Haplotype analyses were further conducted to evaluate the combined effect of the three polymorphisms on OSA risk. As shown in Fig. 2, there were six common haplotypes (>1%) among both cases and controls. The distribution of different haplotypes was similar between OSA patients and controls (Table 3). The most common haplotype was the *-460T/+405C/+936C* (CCT) with frequencies of 29.2% in OSA patients and 44.8% in controls. Associations of OSA risk with *VEGF* haplotypes were shown in Table 3. Compared with haplotype *CCT*, haplotype *CGC* (OR = 2.22, 95% CI = 1.19–4.13) and haplotype *TGC* (OR = 3.83, 95% CI = 1.56–9.40) were associated with a significantly increased risk of OSA.

**Table 3:** Associations between VEGF haplotype and OSA risk.

	Frequency (%)			
Haplotype(–460/+405/936)	Patient	Control	OR (95% CI)	*P*-value
CCT	44 (29.2)	101 (44.8)	1 (Ref.)	
CGC	29 (19.1)	30 (13.5)	2.22 [1.19, 4.13]	0.01
CGT	43 (28.8)	60 (26.5)	1.65 [0.97, 2.79]	0.06
TCT	9 (5.9)	9 (3.9)	2.30 [0.85, 6.17]	0.10
TGC	15 (9.7)	9 (3.9)	3.83 [1.56, 9.40]	0.003
TGT	10 (6.5)	17 (7.5)	1.48 [0.63, 3.49]	0.37

VEGF  =  Vascular endothelial growth factor; OSA  =  Obstructive sleep apnea; OR  =  Odds ratio; CI  =  Confidence interval; Ref.  =  Reference.

### The relationship between *VEGF* genotypes and obesity indexes

Considering obesity was an important risk factor for OSA, we further studied whether *VEGF* genotypes related to BMI or neck circumference in OSA patients. However, no significant association was observed between BMI and genotypes of three *VEGF* polymorphisms (Table 4). None the less, there was no significant difference in neck circumference among patients with different *VEGF* genotypes.

**Table 4:** Comparison of obesity indexes among patients with different genotypes.

Polymorphism	*n*	BMI (kg/m^2^)	Neck Circumference (cm)
VEGF -460C/T			
TT	76	27.1±3.8	40.9±4.2
TC	58	27.6±3.4	39.9±4.3
CC	16	26.8±2.9	41.0±5.6
* P*-Value		0.632	0.449
VEGF +405C/G			
GG	63	26.9±3.2	40.9±3.8
GC	65	27.8±3.7	40.0±5.0
CC	22	26.2±4.1	41.0±4.1
* P*-Value		0.14	0.396
VEGF +936C/T			
CC	94	27.2±3.6	40.1±4.5
CT	46	27.4±3.5	41.0±4.4
TT	10	26.9±3.8	42.0±3.5
* P*-Value		0.926	0.261

## Discussion

In the present study, we evaluated the potential association of *VEGF -460T/C*, *+405G/C*, and *+936C/T* polymorphisms and OSA risk in Chinese population. In addition, we inferred *VEGF -460/+405/+936* haplotypes and compared their frequency distributions in OSA patients and controls. This is the first case-control study, till date, to investigate *VEGF* polymorphisms and haplotypes in relation to OSA, to the best of our knowledge.

Our study showed that the variant allele frequencies of *-460C*, *+405G*, and *+936T* in the control group were 0.18, 0.52, and 0.16, respectively, which were in close agreement with previously published reports of healthy Chinese individuals (0.26, 0.60, and 0.19, respectively) [26], [27]. In our study, a strong linkage disequilibrium was observed for *+405G/C* and *+936C/T*, but its much weaker for *-460T/C* and *+936C/T*. Six common haplotypes were identified to account for >99% of all haplotypes tagged by these three SNPs of *VEGF*. The most common haplotype in the control group of our study was the -*460T/+405C/+936C* (CCT). However, Kataoka and colleague showed different results possibly because only female population was included in their study [20].

The results from our study showed that the three *VEGF* SNPs (*-460T/C*, *+405G/C*, and *+936C/T*) were associated with OSA susceptibility. The *-460C* allele (C *vs.* T: OR = 1.95, 95% CI = 1.38–2.76) and *+936T* allele (T *vs.* C: OR = 1.48, 95% CI = 1.02–2.15) were associated with an increased OSA risk, whereas *+405C* allele was associated with a decreased susceptibility to OSA (C *vs.* G: OR = 0.61, 95% CI = 0.45–0.83). Haplotype analyses were further performed to evaluate the combined effect of the three polymorphisms on OSA risk. Compared with the most common haplotype *CCT*, haplotype *CGC* (OR = 2.22, 95% CI = 1.19–4.13) and *TGC* (OR = 3.83, 95% CI = 1.56–9.40) were associated with a significantly increased risk of OSA. The results suggest that *VEGF* genotypes contributed to an inherited predisposition to OSA. These three *VEGF* SNPs could be used as risk factors for OSA. Moreover, such evidence might allow the identification of higher risk OSA patients in which an intensive treatment with antiangiogenic agents could be of benefit. However, larger prospective and multiethnic studies should be conducted to confirm our findings.

The exact mechanisms of how these polymorphisms modified the risk of OSA remains unclear. Firstly, a possible explanation could be that functional polymorphisms of *VEGF* gene affected its protein expression leading to difference in VEGF-mediated angiogenesis in hypoxic tissues. The levels of VEGF in peripheral blood were significantly higher among OSA patients than those in healthy controls (168.2 pg/ml *vs.* 89.1 pg/ml) [5]. Kaczmarek *et al*. demonstrated an evaluated VEGF expression in skin biopsies of OSA patients with severe nocturnal hypoxemia [28]. None the less, the investigators found a positive correlation between the levels of serum VEGF and AHI (r = 0.34, *P*<0.05) [29]. Secondly, systemic and airway inflammation in OSA could also explain by the elevation of VEGF [30]. It was reported that sputum VEGF and airway vascular permeability index were significantly related to OSA severity and sputum neutrophil number [30]. Thirdly, VEGF might have an effect on upper airway size or function and thus contributed to the susceptibility to the risk of OSA. However, up to data, no publications were performed on this content and it should be further clarification in well-designed, adequately powered studies.

Some limitations of this study should be acknowledged. Firstly, we only investigated three functional polymorphisms of the *VEGF* gene. The association between other polymorphisms in the *VEGF* gene and its relationship to OSA susceptibility requires further study. Secondly, the sample size in this case-control study was not large enough. Therefore, additional studies with larger sample sizes are required to confirm our findings. Thirdly, the serum concentrations of VEGF were not measured in our study, which limited our further investigate whether the levels of serum VEGF correlated with genotypes and cardiovascular co-morbidities.

In summary, this is the first report to our knowledge of the associations between VEGF polymorphisms with the risk of OSA. The *-460C* allele, *+936T* allele, haplotype *CCT*, and haplotype *CGC* were associated with an increased OSA risk, whereas *+405C* allele was associated with a decreased susceptibility to OSA. These results need to be validated by other independent studies, and further studies are necessary to investigate the relationship between *VEGF* polymorphisms and OSA risk in diverse ethnic populations.
